# Statistical considerations for the platform trial in COVID-19 vaccine priming and boosting

**DOI:** 10.1186/s13063-024-08343-y

**Published:** 2024-07-26

**Authors:** Michael Dymock, Charlie McLeod, Peter Richmond, Tom Snelling, Julie A. Marsh, On behalf of the PICOBOO Investigator Team

**Affiliations:** 1 Wesfarmers Centre of Vaccines and Infectious Diseases, Telethon Kids Institute, 15 Hospital Avenue, Nedlands, 6009 Perth, Australia; 2 Infectious Diseases Department, Perth Children’s Hospital, 15 Hospital Avenue, Nedlands, 6009 Perth, Australia; 3 School of Medicine, The University of Western Australia, 35 Stirling Highway, Crawley, 6009 Perth, Australia; 4 Centre for Child Health Research, The University of Western Australia, 35 Stirling Highway, Crawley, 6009 Perth, Australia; 5 General Paediatrics and Immunology Departments, Perth Children’s Hospital, 15 Hospital Avenue, Nedlands, 6009 Perth, Australia; 6 Sydney School of Public Health, Faculty of Medicine and Health, University of Sydney, Camperdown, 2006 Sydney, Australia

**Keywords:** COVID-19, Vaccine, Adaptive trial, Bayesian, Immunogenicity, Reactogenicity

## Abstract

The Platform trial In COVID-19 priming and BOOsting (PICOBOO) is a multi-site, adaptive platform trial designed to generate evidence of the immunogenicity, reactogenicity, and cross-protection of different booster vaccination strategies against severe acute respiratory syndrome coronavirus 2 (SARS-CoV-2) and its variants, specific for the Australian context. The PICOBOO trial randomises participants to receive one of three COVID-19 booster vaccine brands (Pfizer, Moderna, Novavax) available for use in Australia, where the vaccine brand subtypes vary over time according to the national vaccine roll out strategy, and employs a Bayesian hierarchical modelling approach to efficiently borrow information across consecutive booster doses, age groups and vaccine brand subtypes. Here, we briefly describe the PICOBOO trial structure and report the statistical considerations for the estimands, statistical models and decision making for trial adaptations. This paper should be read in conjunction with the PICOBOO Core Protocol and PICOBOO Sub-Study Protocol 1: Booster Vaccination. PICOBOO was registered on 10 February 2022 with the Australian and New Zealand Clinical Trials Registry ACTRN12622000238774.

## Introduction

The coronavirus disease of 2019 (COVID-19) pandemic, caused by severe acute respiratory syndrome coronavirus-2 (SARS-CoV-2), continues to impact global health [1]. Uncertainties about the optimal strategies for COVID-19 priming and booster vaccination remain, including how vaccination impacts key elements of systemic and mucosal immunity and how these immune responses correlate with protection against infection and disease in different populations, especially against future variants of concern (VoC) [2].

Platform trials are increasingly being employed in comparative effectiveness studies for COVID-19 prevention and treatment strategies [3]. Platform designs, incorporating pre-specified trial adaptations, are more flexible and can be more resource efficient than conventional fixed designs. This is due to the repeated scheduled analyses and the potential to stop recruitment early for superiority or futility; further efficiencies are possible using Bayesian methods to share information across multiple participant populations (e.g. across participant age groups), and documentation is under a single core protocol [4–6]. The Platform trial In COVID-19 priming and BOOsting (PICOBOO) is a multi-site, randomised, platform trial that is designed to perpetually allow enrolment for as long as is needed to generate high-quality data to inform National COVID-19 priming and booster vaccination practice and policy [7] (Australian and New Zealand Clinical Trials Registry ACTRN12622000238774). Flexibility is a key requirement of the trial design due to age-related eligibility for access to immunisation as part of the national roll out of COVID-19 vaccines and the emerging VoC. The primary objective of the PICOBOO trial is to estimate the immunological responses induced by different vaccine interventions with sufficient (prespecified) precision. As such, there will be no formal statistical comparisons made between vaccine interventions.

This paper summarises the statistical considerations for the PICOBOO trial and should be read in conjunction with the PICOBOO Core Protocol [7] and the PICOBOO Sub-Study Protocol 1: Booster Vaccination documents. This document is intended to supplement the study protocols and does not replace a formal statistical analysis plan, which will be produced and made available at a later date. We begin by briefly describing the trial structure before specifying the trial subpopulations, randomisation methods and estimands. The Bayesian hierarchical modelling approach used to borrow information across consecutive booster doses, participant age groups and vaccine brand subtypes follows [8, 9], along with a description of the planned trial adaptations and the decision criteria, evaluated at each scheduled analysis [10]. The target populations, endpoints, statistical methods and models and population level estimators are defined within an estimands framework (ICH E9 (R1)) [11]. We conclude with a summary of the design at trial commencement and a discussion on how the PICOBOO trial compares to other innovative contemporaneous designs.

## Trial structure

The PICOBOO adaptive platform trial is initially designed as a three-arm parallel group adaptive trial, corresponding to the three initial SARS-CoV-2 vaccine brands, for either the 1st, 2nd or 3rd booster vaccine dose, with strata based on the combination of primary vaccine history (vaccine brand received for a participant’s priming schedule, i.e. first two doses) and age group. It has the capacity to accommodate additional strata, vaccine brands, vaccine brand subtypes (vaccine subtypes produced by the same manufacturer) and booster vaccine doses or schedules, as both novel vaccines and policy evolves over time in response to emerging VoC. We define, in detail, the notation for the participants, strata, interventions, booster vaccine dose numbers and covariates in the following sections.

### Participants

Let *N* be the number of participants included in a given analysis where participants are denoted by $i \in \varvec{I} = \{1,2,\ldots ,N\}$. Recruited participants may be re-randomised to receive a subsequent booster vaccine dose and may, therefore, contribute repeated measurements to the study.

### Strata: primary vaccine history and age group

Primary vaccine history is denoted by $j \in \varvec{J} = \{\text {AZ, Pf, Mod}\}$, and age is denoted by $l \in \varvec{L} = \{<\text {12y}, 12\text {y to}<18\text {y, 18y to}<50\text {y, 50y to}<70\text {y, }\ge \text {70y}\}$. Here, AZ, Pf and Mod represent the vaccine brand a participant received for their priming schedule (i.e. first two doses) and refer to AstraZeneca’s Vaxzevria (AZD1222), Pfizer BioNTech’s Comirnaty (BNT162b2) and Moderna’s Spikevax (mRNA-1273), respectively, corresponding to available priming COVID-19 vaccine schedules in Australia prior to 2023. Strata are defined as mutually exclusive groups based on the combination of the participant primary vaccine history and age group. Low vaccine uptake was observed for the Pf priming schedule in individuals aged $\ge 70\text {y}$, for the AZ priming schedule in individuals aged $<50\text {y}$ and for the Mod priming schedule in individuals aged $\ge 18\text {y}$, due to Australian vaccine policies in place between 2020 and 2022; therefore, these individuals are ineligible for recruitment into the trial. Additional strata are able to be included as part of the design as the trial progresses.

### Interventions

Separate vaccine brands are denoted by $v \in \{1,2,\ldots ,V\}$. Vaccine brand *v* has subtypes (vaccine subtypes produced by the same manufacturer) $s_v \in \{1,2,\ldots ,S_v\}$, where $S_v$ is the total number of subtypes for vaccine brand *v*. Note that $s_v = 1$ denotes the ancestral subtype for each vaccine brand (i.e. the original vaccine formulation containing the ancestral strain). Separate vaccine interventions are denoted:

$$\begin{aligned} k \in \varvec{K} = \{(v, s_v)\} = \{(1,1),(1,2),\ldots ,(1,S_1),(2,1),(2,2),\ldots ,(2,S_2),\ldots ,(V,1),(V,2),\ldots ,(V,S_V)\} \end{aligned}$$

The trial commenced with Pfizer BioNTech’s Comirnaty (BNT162b2) (Pf), Moderna’s Spikevax (mRNA-1273) (Mod) and Novavax’s Nuvaxovid (NVX-CoV2373) (Nvx) vaccine brands, each with their respective ancestral subtype. At any time, there will only be up to three vaccine interventions allocated in the trial, where each allocation is for the most recently released brand subtype (i.e. allocations to ancestral vaccines cease when the next VoC vaccine, BA.1, becomes available). This labelling will also easily accommodate new interventions should these options expand over time, whether these are vaccine brands or vaccine brand subtypes.

### SARS-CoV-2 booster vaccine dose number

Separate booster vaccine doses are denoted by $m \in \varvec{M}_i \subseteq \varvec{M} = \{1,2,\ldots ,M^*\}$, where $M^*$ is the maximum number of booster doses available for any participant included in a given analysis (note that $M^*$ is known at the time of an analysis and may change as the trial progresses and vaccine policy evolves). We make this distinction clear as participants may be re-randomised into the trial and consequently have varying numbers of observations. For example, participant *i* may enter the trial only for their first booster dose and participant $i'$ may enter the trial for their second booster dose and consent to be re-randomised for their third booster dose. In this case, $\varvec{M}_i = \{1\}$ and $\varvec{M}_{i'} = \{2,3\}$. This labelling will also easily accommodate further booster vaccine doses, and skipped randomised booster dose occasions, should these options expand over time.

### Covariates

Participant *i*’s covariates for their $m^\text {th}$ booster vaccine dose are denoted $\varvec{x}_{im} = \{x_{im1}, x_{im2},\ldots ,x_{imP}\}$ and are governed by *P* model parameters. The covariates for the primary model include standardised $\text {log}_{10}$ anti-spike SARS-CoV-2 immunoglobulin G (IgG) concentration immediately prior to vaccine administration for current randomisation occasion, previous COVID-19 infection (defined below), site and sex. Continuous covariates are standardised within stratum and booster dose number and the reference value for categorical covariates is set to the most frequently observed. The covariates may differ for the secondary models.

#### Previous COVID-19 infection

The covariate *previous COVID-19 infection* is derived for the current randomisation occasion using participant reported previous SARS-CoV-2 infection in combination with their baseline anti-nucleocapsid antibodies test result, where a positive result indicates previous infection. The details for this derivation are in Table 1. Note that in the rare scenario where a participant is missing a value for their reported previous SARS-CoV-2 infection, it is presumed (conservatively) that they have have not been previously infected unless there is evidence indicating otherwise (e.g. via a positive baseline anti-nucleocapsid antibodies test result).

**Table 1:** Derivation of previous COVID-19 infection covariate

Participant reported previous SARS-CoV-2 infection	Baseline anti-nucleocapsid antibodies test result	Derived variable
No	Negative	No
	Positive	Yes
	Missing	No
Yes	Negative	Yes
	Positive	Yes
	Missing	Yes
Missing	Negative	No
	Positive	Yes
	Missing	No

#### Epochs

We introduce time epochs to address potential concerns regarding the impact of time on a multi-year trial (e.g. evolution of the circulating SARS-CoV-2 variants or the prevalence of COVID-19). We denote participant *i*’s time epoch relative to trial commencement for their $m^\text {th}$ booster vaccine dose as $\varvec{z}_{im} = \{z_{im1}, z_{im2},\ldots ,z_{imQ}\}$, where there are *Q* epochs. At a scheduled analysis, epochs will start at the date of data cut-off and be counted backwards using 6 month periods until the time of trial commencement. Epochs will be modelled using the Bayesian time machine approach demonstrated by Saville et al. [12–14].

## Analysis sets

We define distinct but potentially overlapping analysis sets (trial populations) in order to precisely define the estimands. A summary of the analysis sets can be found in Table 2.

**Table 2:** Summary of analysis sets

Analysis set	Abbreviation	Description
Modified intention-to-treat	MI	All participants who were randomised to an intervention, provided a blood sample within the appropriate estimand window and do not have evidence of receiving a further SARS-CoV-2 vaccine dose between randomisation and the time of endpoint. Participants will be analysed according to their randomised intervention irrespective of withdrawal, treatment compliance or other protocol deviations
Modified intention-to-treat C7	MI-C7	Subset of MI without evidence of a SARS-CoV-2 infection between randomisation and provision of a $\sim 7$ day blood sample after trial vaccine dose
Modified intention-to-treat C28	MI-C28	Subset of MI without evidence of a SARS-CoV-2 infection between randomisation and provision of a $\sim 28$ day blood sample after trial vaccine dose
Immunological subset C7	IS-C7	Subset of MI-C7 who were sequentially enrolled into the study for additional detailed laboratory analysis
Immunological subset C28	IS-C28	Subset of MI-C28 who were sequentially enrolled into the study for additional detailed laboratory analysis
Safety population	SP	All participants who were randomised to, and received, an intervention. Participants will be analysed according to the intervention received, irrespective of withdrawal or other protocol deviations

### Modified intention-to-treat

The modified intention-to-treat (MI) analysis set includes all participants randomised to an intervention that provided a blood sample within the appropriate window for endpoint collection and do not have evidence of receiving a further SARS-CoV-2 vaccine dose between randomisation and the time of endpoint.

#### Modified intention-to-treat without SARS-CoV-2 infection

We define a subset of the MI analysis set with those participants without evidence of a SARS-CoV-2 infection between randomisation and provision of a $\sim 7$ day blood sample after trial vaccine dose. We denote this analysis set MI-C7. We define another subset of the MI analysis set with those participants without evidence of a SARS-CoV-2 infection between randomisation and provision of a $\sim 28$ day blood sample after trial vaccine dose. We denote this analysis set MI-C28. Evidence of SARS-CoV-2 infection after randomisation includes a rapid antigen test (RAT) or polymerase chain reaction (PCR) confirmed reported infection or a positive anti-nucleocapsid antibodies test for participants with a negative anti-nucleocapsid antibodies test at baseline.

### Immunological subset

A subset of sequentially enrolled participants will be recruited from selected sites to provide blood samples for additional detailed laboratory analysis of markers of vaccine immune responses. In particular, the first 20 participants receiving each intervention, for each booster dose number in each stratum to provide a sample at visit 3 (day 28) within the visit window, will be included in the immunological subset. Participants who incorrectly receive a vaccine that does not match their allocated vaccine will be ineligible for the immunological subset.

#### Immunological subset without SARS-CoV-2 infection

We define subsets of the MI-C7 and MI-C28 trial populations with those participants that are members of the immunological subset. We denote these subsets IS-C7 and IS-C28, respectively.

### Safety population

In safety population (SP) analyses, all randomised participants who received a vaccine will be analysed according to the intervention they received. Participants who do not receive a vaccine will be excluded from the SP set, whereas trial-ineligible participants who are incorrectly randomised and received an intervention will be included in the SP.

## Randomisation

Enrolled participants will be randomly allocated to one of the available interventions with equal allocation probabilities. Randomisation is subject to random permuted blocks and is stratified by stratum and booster dose number. Re-randomised participants have their subsequent randomisation additionally stratified by the first trial intervention they were allocated to. New vaccines will replace existing interventions as the trial progresses and available vaccines reflect the circulating variants (e.g. when a bivalent formulation supersedes the ancestral formulation within a vaccine brand).

## Statistical modelling

Bayesian statistical methods for clinical trials are increasingly being used over the classical frequentist approach [15]. These methods allow us to incorporate the subjective previous knowledge of intervention effects (via a prior distribution) with the observed data to produce an updated state of knowledge (a posterior distribution). The PICOBOO adaptive trial employs Bayesian hierarchical methods in order to efficiently share information, accrued from observed data and prior knowledge, between estimates for consecutive booster vaccine doses, age groups and interventions. We detail the estimands and Bayesian models, including the prior distributions for the model parameters, in the following sections.

### Estimands

The estimands are summarised in Table 3 with further detail provided in the core protocol [7]. The estimands include continuous, percentage, count and binary outcomes.

**Table 3:** Summary of trial estimands

ID	Analysis set	Outcome	Time (days)
01	MI-C28	Ancestral SARS-CoV-2 anti-spike IgG concentration	28 (21–31)
02	MI-C7	Ancestral SARS-CoV-2 anti-spike IgG concentration	7 (6–8)
03-05	MI-C28	Ancestral SARS-CoV-2 anti-spike IgG concentration	84 (70–98), 180^a^ (152–208) and 365 (337–393)
06-10	MI	Ancestral SARS-CoV-2 anti-spike IgG concentration	7 (6–8), 28 (21–31), 84 (70–98), 180 (152–208) and 365 (337–393)
11-14	IS-C28	Ancestral SARS-CoV-2 neutralising antibodies concentration	28 (21–31), 84 (70–98), 180 (152–208) and 365 (337–393)
15-18	IS-C28	SARS-CoV-2 predominant circulating variant^b^ neutralising antibodies concentration	28 (21–31), 84 (70–98), 180 (152–208) and 365 (337–393)
19-22	IS-C28	Ancestral SARS-CoV-2 percentage inhibition of virus	28 (21–31), 84 (70–98), 180 (152–208) and 365 (337–393)
23-26	IS-C28	SARS-CoV-2 predominant circulating variant percentage inhibition of virus	28 (21-31), 84 (70-98), 180 (152-208) and 365 (337-393)
27	IS-C7	Number of IFN-$\gamma$ spot forming cells per $10^6$ peripheral blood mononuclear cells following stimulation with ancestral SARS-CoV-2 spike overlapping pools of lyophilized peptides, consisting mainly of 15-mer sequences with 11 amino acids overlap	7 (6-8)
28	IS-C28	Number of IFN-$\gamma$ spot forming cells per $10^6$ peripheral blood mononuclear cells following stimulation with ancestral SARS-CoV-2 spike overlapping pools of lyophilized peptides, consisting mainly of 15-mer sequences with 11 amino acids overlap	28 (21-31)
29	IS-C7	Number of IFN-$\gamma$ spot forming cells per $10^6$ peripheral blood mononuclear cells following stimulation with SARS-CoV-2 predominant circulating variant spike overlapping pools of lyophilized peptides, consisting mainly of 15-mer sequences with 11 amino acids overlap	7 (6-8)
30	IS-C28	Number of IFN-$\gamma$ spot forming cells per $10^6$ peripheral blood mononuclear cells following stimulation with SARS-CoV-2 predominant circulating variant spike overlapping pools of lyophilized peptides, consisting mainly of 15-mer sequences with 11 amino acids overlap	28 (21-31)
31	MI-C7	Ancestral SARS-CoV-2 serum anti-spike IgG concentration	7 (6-8)
32-35	MI-C28	Ancestral SARS-CoV-2 serum anti-spike IgG concentration	28 (21–31), 84 (70–98), 180 (152–208) and 365 (337–393)
36	MI-C7	SARS-CoV-2 predominant circulating variant serum anti-spike IgG concentration	7 (6–8)
37-40	MI-C28	SARS-CoV-2 predominant circulating variant serum anti-spike IgG concentration	28 (21–31), 84 (70–98), 180 (152–208) and 365 (337–393)
41	SP	Serious adverse events suspected to be related to vaccine booster in the 31 days following randomisation	28 (21–31)

^a^Adolescents and participants randomised after January 30, 2023, will provide blood samples at day 180 instead of day 84 for visit 4. Participants that have been re-randomised will provide a blood sample and receive their subsequent dose at day 180. Adolescents will not have blood samples collected at day 365

^b ^The predominant circulating variant(s) will be determined independently at each scheduled analysis and may vary over the course of the trial

### Planned exploratory analyses

In addition to the estimands in Table 3, we specify a series of planned exploratory analyses that are contingent on laboratory capacity and resource availability. Similar to estimands 28 and 30, we may also analyse the number of interferon-gamma (IFN-${\gamma }$) spot forming cells per $10^6$ peripheral blood mononuclear cells following stimulation with ancestral and predominant circulating variant SARS-CoV-2 spike overlapping pools of lyophilized peptides, consisting mainly of 15-mer sequences with 11 amino acids overlap, at 84 (70–98) days, 180 (152–208) days and 365 (337–393) days after randomisation, in the IS-C28 analysis set. Additionally, for the MI-C28 analysis set, we may analyse ancestral and predominant circulating variant SARS-CoV-2 mucosal salivary anti-spike immunoglobulin A (IgA) and IgG concentrations at 28 (21–31) days, 84 (70–98) days, 180 (152–208) days and 365 (337–393) days after randomisation.

Furthermore, as the trial progresses, new and emerging laboratory tests and procedures may supersede those currently stated and existing tests may be removed if they are deemed unreliable or uninformative.

### Descriptive statistics for demographic variables and safety outcomes

Demographic data summarised by stratum, booster dose number and intervention will be presented for each estimand. The demographic variables will include, but may not be restricted to, age, sex, ethnicity and comorbidity status. Continuous variables will be summarised by median and interquartile range and categorical variables will be summarised by frequency and percentage. Safety data will be presented in a separate safety data report and includes tabulated and line listed summaries of solicited reactogenicity data on days 1–7 (collected via diary cards), solicited adverse events within 28 days, COVID-19 infections, unsolicited adverse events and serious adverse events.

### Descriptive statistics for immunogenicity outcomes

Descriptive statistics including the geometric mean and mean and standard deviation on the data scale, summarised by stratum, booster dose number and intervention will be presented alongside each corresponding planned analysis. All descriptive statistics will be unadjusted (i.e. not modelled).

### Deviations from the protocol

All deviations from the protocol including missing visits and additional COVID-19 vaccines received will be summarised by stratum, booster dose number and randomised intervention.

### Missing data

Missing outcome data will be assumed missing at random and excluded from analyses (i.e. a complete case strategy). Epoch, site and sex covariates consist of critical data and so will not be missing for any participants. The previous COVID-19 infection covariate is derived such that it accounts for missing data and any missing continuous covariates (e.g. baseline immunological data) will be set to the respective stratum and booster dose number standardised mean (i.e. zero).

### General linear function

We define a general linear function that is common to all statistical models (albeit with the appropriate specific linking functions) as follows:

1 $$\begin{aligned} f(i, j, k, l, m) = \mu _{jklm} + \sum \limits _{p=1}^P x_{imp}\beta _{kp} + \sum \limits _{q=1}^Q z_{imq}\gamma _q \end{aligned}$$

Here, $\mu _{jklm}$ is a mean parameter dependent on a participant’s primary vaccine history, intervention received, age group and booster dose number, $\varvec{\beta }_k = \{\beta _{k1},\beta _{k2},\ldots ,\beta _{kP}\}$ is the parameter vector governing the covariates for intervention *k* and $\gamma _q$ is the parameter for the effect of the $q^\text {th}$ epoch (i.e. $\gamma _1$ is the most recent epoch).

### Primary model

The primary model will be used for the analysis of estimands 01, 02, 03–05, 06–10, 11–14, 15–18, 31, 32–35, 36 and 37–40 in Table 3. A Bayesian three-level hierarchical model will be used as it is anticipated that immune responses may be mutually informative across SARS-CoV-2 booster dose number, age groups and potentially across vaccine brands using mRNA technology (Pf and Mod). However, prior distributions have been chosen to ensure that the level of information sharing is data driven [8, 9]. In addition, the covariates included in the model and the parameters for the prior distributions will be chosen specific to the endpoint to ensure numerical stability and scientific appropriateness. The model estimates the posterior distribution of the mean $\text {log}_{10}$ concentration for each intervention and booster dose number in each stratum.

We model the continuous endpoints for participant *i*, denoted $\varvec{Y}_{ij\varvec{k^*} l} \in \mathbb {R}^{|\varvec{M}_i|} \subseteq \mathbb {R}^{M^*}$, with a hierarchical linear model with normally distributed residuals, where $|\varvec{M}_i|$ is the number of outcomes for participant *i*, noting that a participant will have multiple outcomes if and only if they consent to be re-randomised to subsequent booster doses. For example, a participant that receives their first booster dose and is re-randomised to a second booster dose will have a two-dimensional outcome: $$\varvec{Y}_{ij\varvec{k^*} l} = \left( \begin{array}{c} Y_{ijkl1} \\ Y_{ijk'l2} \end{array}\right) \in \mathbb {R}^2$$. Here, $\varvec{k^*} = \{k,k'\} \subseteq \varvec{K}$ as a re-randomised participant may receive the same or different vaccine interventions at each randomisation.

We denote $\varvec{\Sigma }_l$ as a $M^*\times M^*$ symmetric positive definite matrix with diagonal elements $\Sigma _{l[mm]} = \sigma ^2$, i.e. for the $m^\text {th}$ row and column, and off diagonal elements $\Sigma _{l[mm']} = r_{lmm'}\sigma ^2$, i.e. for the $m^\text {th}$ row and ${m'}^\text {th}$ column (note that $r_{lmm'} = r_{lm'm}$ by symmetry). Recalling that $|\varvec{M}_i|$ is the number of outcomes for participant *i*, we introduce the notation $[\cdot ]^{|\varvec{M}_i|}$ and $[\cdot ]^{|\varvec{M}_i| \times |\varvec{M_i}|}$ to denote a vector of length $|\varvec{M}_i|$ and a matrix with dimension $|\varvec{M}_i| \times |\varvec{M}_i|$, respectively. For example, $[f(i,j,\varvec{k^*},l,m)]^{|\varvec{M}_i|}$ is a $|\varvec{M}_i|$ length vector containing the linear predictor evaluated for each of participant *i*’s outcomes and $[\varvec{\Sigma }_l]^{|\varvec{M}_i| \times |\varvec{M}_i|}$ is the appropriate $|\varvec{M}_i| \times |\varvec{M}_i|$ dimension submatrix of the full covariance matrix relevant for participant *i*. This notation allows the model to accommodate participants with different numbers of outcomes corresponding to the different randomisation occasions. The primary model is then:

2 $$\begin{aligned} & \varvec{Y}_{ij\varvec{k^*} l} \sim \text {N}\left( \left[f(i,j,\varvec{k^*},l,m)\right]^{|\varvec{M}_i|}, [\varvec{\Sigma }_l]^{|\varvec{M}_i| \times |\varvec{M}_i|}\right) \nonumber \\ & \forall i \in \varvec{I}, j \in \varvec{J}, \varvec{k^*} \subseteq \varvec{K}, l \in \varvec{L}, m \in \varvec{M} \end{aligned}$$

The three level hierarchical structure on the prior distribution for $\mu _{jklm}$ is as follows:

First level (leverage information across booster dose numbers):

3 $$\begin{aligned} \mu _{jklm} \sim \text {N}\left(\mu _{jkl}, \tau ^2_{jkl}\right) \quad \forall j \in \varvec{J}, k \in \varvec{K}, l \in \varvec{L}, m \in \varvec{M} \end{aligned}$$

Second level (leverage information across age groups):

4 $$\begin{aligned} \mu _{jkl} \sim \text {N}\left(\mu _{jk}, \tau ^2_{jk}\right) \quad \forall j \in \varvec{J}, k \in \varvec{K}, l \in \varvec{L} \end{aligned}$$

Third level (leverage information across mRNA vaccine interventions):

5 $$\begin{aligned} \mu _{jk} \sim \text {N}\left(\mu _j, \tau ^2_j\right) \quad \forall j \in \varvec{J}, k \in \{k \in \varvec{K} | v \in \{\text {Pf}, \text {Mod}\}\} \end{aligned}$$

The hyperprior distributions are:

6 $$\begin{aligned} & \mu _{jk} \sim \text {N}\left( \text {log}_{10}(8,347), 0.5^2\right) \quad \forall j \in \varvec{J}, k \in \{k \in \varvec{K} | v = \text {Nvx}\} \end{aligned}$$

7 $$\begin{aligned} & \mu _j \sim \text {N}\left( \text {log}_{10}(20,517), 0.5^2\right) \quad \forall j \in \varvec{J} \end{aligned}$$

8 $$\begin{aligned} & \tau _{jkl}, \tau _{jk}, \tau _j \sim \text {IG}(3,1) \quad \forall j \in \varvec{J}, k \in \varvec{K}, l \in \varvec{L} \end{aligned}$$

The prior means in Eqs. 6 and 7 are based on data from the Comparing COVID-19 booster vaccinations (COV-BOOST) trial publication [16] for ChAdOx1 nCov-19 vaccine homologous priming with one booster of NVX-CoV2373 vaccine/Novavax (8,347 ELU/ml) and BNT162b2 vaccine/Pfizer-BioNTech (20,517 ELU/ml). Note that these prior means and standard deviations are specific for the analysis of estimand 01 and may vary for analyses of other continuous endpoints, which will be pre-specified in statistical analysis plans. The priors for the covariate terms are:

9 $$\begin{aligned} \beta _{kp} \sim \text {N}(0,1) \quad \forall k \in \varvec{K}, p \in \{1,2,\ldots ,P\} \end{aligned}$$

We implement a time machine approach for the epoch parameters following the approach described in Mahar et al. [14]. We define the first-order dynamic model with prior distributions:

10 $$\begin{aligned} \gamma _1 & = 0 \end{aligned}$$

11 $$\begin{aligned} \gamma _q & \sim \text {N}\left(\gamma _{q-1}, \omega _q^2\right) \quad \forall q \in \{2, 3,\ldots ,Q\} \end{aligned}$$

12 $$\begin{aligned} \omega _q & \sim \text {IG}(3,1) \quad \forall q \in \{2, 3,\ldots ,Q\} \end{aligned}$$

We then impose a prior structure on the *decomposed* covariance matrix, where $\varvec{Q}_l$ contains the standard deviation parameters ($\sigma _l$), $\varvec{I}_{M^*}$ is an $M^*$-dimensional identity matrix and $\varvec{R}_l$ contains the correlation parameters ($r_{lmm'}$):

13 $$\begin{aligned} \varvec{\Sigma }_l & = \varvec{Q}_l\varvec{R}_l\varvec{Q}_l \quad \forall l \in \varvec{L}\end{aligned}$$

14 $$\begin{aligned} \varvec{Q}_l & = \sigma _l\varvec{I}_{M^*} \quad \forall l \in \varvec{L}\end{aligned}$$

15 $$\begin{aligned} \varvec{R}_l & = \left( \begin{array}{ccc} 1 & \ldots & r_{l1M^*} \\ \vdots & \ddots & \vdots \\ r_{lM^*1} & \ddots & 1 \end{array}\right) \quad \forall l \in \varvec{L}\end{aligned}$$

16 $$\begin{aligned} \sigma _l & \sim \text {Exponential}(0.5) \quad \forall l \in \varvec{L} \end{aligned}$$

17 $$\begin{aligned} \varvec{R}_l & \sim \text {LKJcorr}(2) \quad \forall l \in \varvec{L} \end{aligned}$$

### Percentage inhibition of virus endpoint model

A Bayesian beta regression model with a logistic link function will be used for all percentage inhibition of virus endpoints, by first converting the outcomes to a continuous proportion by dividing by one hundred (see estimands 19–22 and 23–26 in Table 3). The model estimates the posterior distribution of the mean proportion inhibition of virus after randomisation for each intervention and booster dose number in each stratum. We model the proportion inhibition of virus for participants, where $Y_{ijklm} \in [0,1]$, using the following Bayesian beta regression model:

18 $$\begin{aligned} & \begin{array}{l} Y_{ijklm} \sim \text {Beta}\left( \eta _{ijklm}\phi _{jklm}, (1-\eta _{ijklm})\phi _{jklm}\right) \\ \forall i \in \varvec{I}, j \in \varvec{J}, k \in \varvec{K}, l \in \varvec{L}, m \in \varvec{M} \end{array} \end{aligned}$$

19 $$\begin{aligned} & \text {logit}(\eta _{ijklm}) = f(i,j,k,l,m) \quad \forall i \in \varvec{I}, j \in \varvec{J}, k \in \varvec{K}, l \in \varvec{L}, m \in \varvec{M} \end{aligned}$$

20 $$\begin{aligned} & \text {log}(\phi _{jklm}) = \psi _{jklm} \quad \forall j \in \varvec{J}, k \in \varvec{K}, l \in \varvec{L}, m \in \varvec{M} \end{aligned}$$

The beta model is parameterised in this way so that $\eta _{ijklm} \in (0,1)$ is the mean of the distribution and $\phi _{jklm} > 0$ is a dispersion parameter (high values of $\phi _{jklm}$ represent low dispersion). Here, the linear predictor enters the model via a logistic link on $\eta _{ijklm}$. The prior distributions imposed on $\mu _{jklm}$ and $\psi _{jklm}$ are:

21 $$\begin{aligned} \mu _{jklm} & \sim \text {N}\left(0, 2^2\right) \quad \forall j \in \varvec{J}, k \in \varvec{K}, l \in \varvec{L}, m \in \varvec{M} \end{aligned}$$

22 $$\begin{aligned} \psi _{jklm} & \sim \text {N}(0, 1) \quad \forall j \in \varvec{J}, k \in \varvec{K}, l \in \varvec{L}, m \in \varvec{M} \end{aligned}$$

The parameters $\beta _{kp}$ and $\gamma _q$ will have the same prior distributions as in Eqs. 9 and 11.

### Count endpoint model

A Bayesian Poisson regression model with a log link function will be used for all count endpoints (see estimands 27, 28, 29 and 30 in Table 3). The model estimates the posterior distribution of the mean rate of the number of IFN-$\gamma$ spot forming cells per $10^6$ peripheral blood mononuclear cells following stimulation with virus after randomisation for each intervention and booster dose number in each stratum. We model the count endpoint for participants, where $Y_{ijklm} \in \mathbb {Z}^+$, using the following Bayesian Poisson regression model:

23 $$\begin{aligned} & Y_{ijklm} \sim \text {Poisson}(\lambda _{ijklm}) \quad \forall i \in \varvec{I}, j \in \varvec{J}, k \in \varvec{K}, l \in \varvec{L}, m \in \varvec{M} \end{aligned}$$

24 $$\begin{aligned} & \text {log}(\lambda _{ijklm}) = f(i,j,k,l,m) \quad \forall i \in \varvec{I}, j \in \varvec{J}, k \in \varvec{K}, l \in \varvec{L}, m \in \varvec{M} \end{aligned}$$

The Poisson model is parameterised in this way so that $\lambda _{ijklm} > 0$ is the mean rate and is connected to the linear predictor via a log link function. A weakly informative normal prior distribution will be imposed on $\mu _{jklm}$, and $\beta _{kp}$ and $\gamma _q$ will have the same prior distributions as in Eqs. 9 and 11.

### Binary endpoint model

A Bayesian logistic regression model will be used for the binary safety endpoint (see estimand 41 in Table 3). The model estimates the posterior distribution of the probability of a serious adverse event after randomisation for each intervention and booster dose number in each stratum. We model the binary endpoint for participants, where $Y_{ijklm} \in \{0,1\}$, using the following Bayesian logistic regression model:

25 $$\begin{aligned} & Y_{ijklm} \sim \text {Bernoulli}(\pi _{ijklm}) \quad \forall i \in \varvec{I}, j \in \varvec{J}, k \in \varvec{K}, l \in \varvec{L}, m \in \varvec{M} \end{aligned}$$

26 $$\begin{aligned} & \text {logit}(\pi _{ijklm}) = f(i,j,k,l,m) \quad \forall i \in \varvec{I}, j \in \varvec{J}, k \in \varvec{K}, l \in \varvec{L}, m \in \varvec{M} \end{aligned}$$

The logistic regression model is parameterised in this way so that $\pi _{ijklm} \in (0,1)$ is the mean probability and is connected to the linear predictor via a logistic link function. A weakly informative normal prior distribution will be imposed on $\mu _{jklm}$, and $\beta _{kp}$ and $\gamma _q$ will have the same prior distributions as in Eqs. 9 and 11.

### Computational methods

All statistical models will be programmed in the probabilistic programming language stan [17]. To interface with stan, we use the cmdstanr package [18] within the R statistical programming environment v4.2.2 [19]. Posterior distributions will be estimated via Markov chain Monte Carlo (MCMC) using stan’s Hamiltonian Monte Carlo algorithm. Each analysis will incorporate eight MCMC chains, run in parallel, with warm-up and sampling phases each running for 1000 iterations. Sampling diagnostics including trace plots, effective sample sizes and divergent transitions will be monitored and assessed to determine algorithm convergence. As appropriate, the team may adjust the sampling specifications accordingly and document this in any arising publications and reports.

Discretion is made for analyses to vary from the detail presented here in order to address model issues. For example, if some model parameters are uninformed due to no participants within a specific category or stratum, then the model may be reparameterised or those parameters may not be reported. Furthermore, in consultation with the DSMC, the analytic team may recommend against conducting a prespecified analysis if there is insufficient data to produce meaningful results.

## Statistical quantities, scheduled analyses and decision rules

The quantities of interest in the PICOBOO trial are the mean $\text {log}_{10}$ ancestral SARS-CoV-2 anti-spike IgG concentration measured $\sim$ 28 days after randomisation for each intervention and booster dose number in each stratum. These quantities will be derived from estimand 01 (Table 3) using the primary model (Eq. 2). Model parameter ($\mu _{jklm}$) posterior distributions will be employed to inform trial adaptation decisions and report to the DSMC, in addition to quantifying intervention effects in any trial publications.

### Scheduled analyses

We define a vaccine booster occasion as any time that a participant is randomised to receive a vaccine booster in the PICOBOO trial. We distinguish between a “vaccine booster occasion” and a “randomised participant” because each participant may contribute multiple vaccine booster occasions if they consent for re-randomisation. The first scheduled analysis will be performed after participants have completed 300 vaccine booster occasions and have completed their $\sim$28 day endpoint post randomisation, and the results from the batched blood samples are available from the laboratory analysis. Thereafter, scheduled analyses will be performed after every 150 additional vaccine booster occasions with available $\sim$28 day laboratory results for the remainder of the trial. Data will be extracted from the PICOBOO database immediately prior to the commencement of each scheduled analysis and the PICOBOO analytic team will prepare a report containing all pre-specified analyses for which the data is available (including immunogenicity and safety data) for the DSMC and trial investigators. The DSMC will make recommendations to the PICOBOO trial steering committee based on an assessment of the report.

### Precision

We will compute the precision of each statistical quantity of interest to be assessed against pre-specified precision criteria. The precision, $\rho _{jklm}$, of the posterior distribution of $\mu _{jklm}$ is defined as the width of $95\%$ highest density credible interval:

27 $$\begin{aligned} \rho _{jklm} = \widehat{\mu _{jklm,U}} - \widehat{\mu _{jklm,L}} \end{aligned}$$

Here, $\widehat{\mu _{jklm,U}}$ and $\widehat{\mu _{jklm,L}}$ represent the upper and lower bounds, respectively, of the $95\%$ highest density credible interval of the posterior distribution of $\mu _{jklm}$. High values of $\rho _{jklm}$ indicate high uncertainty, and therefore low precision, in the estimation of $\mu _{jklm}$. Similarly, low values of $\rho _{jklm}$ indicate low uncertainty, and therefore high precision, in the estimation of $\mu _{jklm}$.

We define the precision criteria for each booster dose number (*m*) in each stratum $(j \times l)$ as:

28 $$\begin{aligned} \rho _{jklm} < 0.2 \quad \forall k \in \varvec{K} \end{aligned}$$

Here, we say that the precision criteria has been met for a booster dose number within a stratum if the width of the $95\%$ highest density credible interval for the mean parameter $\mu _{jklm}$ is less than 0.2 units on the $\text {log}_{10}$ scale for all currently available interventions. Assuming the posterior distribution is approximately symmetric, on the untransformed scale (U/mL), this equates to lower and upper bounds corresponding to a multiplicative reduction of 0.794 or a multiplicative increase of 1.259 to the mean. This threshold was determined through discussions with clinicians in conjunction with extensive computer simulations demonstrating its suitability across a range of plausible trial scenarios [20].

### Trial adaptations

At a scheduled analysis, the precision will be assessed against the precision criteria for each booster dose number in each stratum for estimand 01. If the precision criteria is met, i.e. the precision is sufficiently high, within a booster dose number in a stratum then *recruitment will be ceased* into that booster dose number in that stratum. The outcomes of the precision criteria assessments will be included in the report provided to the trial investigators and DSMC. If the precision criteria is not met, then recruitment will be ceased to a booster dose number within a stratum once there are at least 50 participants randomised to each active intervention.

## Trial commencement

At commencement, the PICOBOO trial enrolled participants over 18 years of age with an AZ or Pf primary vaccine history and randomised each to receive one of the Pf, Mod or Nvx ancestral formulations for their first vaccine booster dose.

To validate the trial design prior to trial commencement, computer simulations were generated to determine the trial operating characteristics under a range of plausible scenarios [20]. The objective of the simulation study was to assess the suitability of the precision threshold (i.e. whether or not adaptations were triggered due to sufficient precision within a booster dose number in a stratum). There were no predefined formal criteria to determine the threshold’s suitability. Trial simulations were explored by varying the number and timing of sequential analyses, precision criteria threshold, recruitment rates and intervention means and standard deviations. The simulations assumed full recruitment up to a maximum of 1000 participants providing 1200 vaccine booster occasions (specified in Table 4) including 5% loss to follow up between 21 and 31 days after randomisation (for estimand 01). We chose mean and standard deviation ancestral SARS-CoV-2 anti-spike IgG concentrations to be similar to those in the COV-BOOST trial publication [16]. The standard deviation for the simulated $\text {log}_{10}$ ancestral SARS-CoV-2 anti-spike IgG concentration was set at 0.3 for the age groups under 70 years of age and 0.4 otherwise. Sensitivity simulations were tested by varying the mean using the respective lower and upper 95% confidence limits and increasing the standard deviation to 0.4 for all age groups.

**Table 4:** Maximum planned recruitment for trial simulation. The number of participants recruited and (consented for re-randomisation)

Vaccine history (*j*)	Age group (*l*)	1st booster dose	2nd booster dose	3rd booster dose
AZ	$\ge \text {70y}$	0	0	150
	$\text {50y to}<\text {70y}$	0	150	50 (100)
Pf	$\text {50y to}<\text {70y}$	0	150	50 (100)
	$\text {18y to}<\text {50y}$	0	150	0
	$\text {12y to}<\text {18y}$	150	0	0
Mod	$\text {12y to}<\text {18y}$	150	0	0

The simulation results including the median precision and proportion of trials where the precision criteria is met are presented in Table 5. The trial operating characteristics were consistent across varying mean scenarios, but, as anticipated, an increase in the standard deviation led to a decrease in the precision. A maximum sample size of 1000 participants, contributing 1200 vaccine booster occasions, allowing for up to 5% loss to follow-up or due to intercurrent COVID-19 infection, is required for at least five cells, consisting of a unique booster dose number and stratum, to meet the precision criteria in over 76% of simulated trials. Furthermore, the precision in the remaining cells approached the precision threshold.

**Table 5:** Proportion of simulated trials with strata meeting the precision criteria and the median precision

Stratum	Pf mean (ELU/mL)	Mod mean (ELU/mL)	Nvx mean (ELU/mL)	Proportion meeting precision criteria^a^	Median precision^b^
AZ $\ge \text {70y}$	19,100	27,700	5,800	0.04	0.22
AZ $\text {50y to}<\text {70y}$	22,500	35,500	8,400	0.89	0.17
Pf $\text {50y to}<\text {70y}$	24,800	44,500	12,600	0.88	0.17
Pf $\text {18y to}<\text {50y}$	24,800	44,500	12,600	0.76	0.17
Pf $\text {12y to}<\text {18y}$	24,800	44,500	12,600	0.80	0.17
Mod $\text {12y to}<\text {18y}$	24,800	44,500	12,600	0.86	0.17

^a^The proportion of trials where the precision criteria has been met for all interventions within a stratum

^b ^Computed at trial conclusion for the earliest booster dose within a stratum across all interventions

## Current state

The PICOBOO trial opened recruitment on 29 March, 2022, and as of the data cut off date for the third scheduled analysis on 29 August, 2023, has recruited 744 participants across three sites in Perth, Adelaide and Launceston, Australia. Participants have been recruited to all strata and booster dose numbers as indicated in Table 4 with the addition of early recruitment to AZ / $\ge 70$/2nd booster dose, AZ/50y to < 70y/1st booster dose, Pf /50y to < 70y/1st booster dose and Pf/18y to < 50y/1st booster dose.

## Discussion

The PICOBOO trial follows in the footsteps of other contemporaneous platform trials, including the Australasian COVID-19 Trial (ASCOT) [21], the Randomized Embedded Multifactorial Adaptive Platform trial for Community-Acquired Pneumonia (REMAP CAP) [22] and the Staphylococcus Network Adaptive Platform trial (SNAP) [14, 23], to pave the way forward for innovative, resource-efficient trial designs in the clinical research space.

This paper provides a detailed account of the statistical considerations for the PICOBOO trial. As the trial progresses, interim statistical reports will be made available online with accompanying statistical implementation guides. The purpose of the statistical implementation guides will be to detail the exact specifications of the trial structure, analysis populations and statistical modelling at the time of each scheduled analysis, in contrast to the more general overview of the statistical components provided here. A full statistical analysis plan will be produced upon trial conclusion to detail the final statistical analysis and will be provided on the trial website.

## Data Availability

Access to data will be granted to study investigators and authorised representatives from the sponsor and the regulatory authorities to allow trial-related monitoring, audits and inspections to occur. PICOBOO will also comply with relevant jurisdictional and academic requirements relating to access to data, as applied at the time that the data are generated.

## Abbreviations

ASCOT: Australasian COVID-19 trial
AZ: AstraZeneca Vaxzevria
COVID-19: Coronavirus 2019
COV-BOOST: Comparing COVID-19 booster vaccinations
DSMC: Data safety and monitoring committee
IFN-$\gamma$: Interferon-gamma
IgA: Immunoglobulin A
IgG: Immunoglobulin G
IS-C28: Immunological subset COVID-19 28 days
IS-C7: Immunological subset COVID-19 7 days
MCMC: Markov chain Monte Carlo
MI: Modified intention-to-treat
MI-C28: Modified intention-to-treat COVID-19 28 days
MI-C7: Modified intention-to-treat COVID-19 7 days
Mod: Moderna Spikevax (mRNA-1273)
mRNA: Messenger ribonucleic acid
Nvx: Novavax Nuvaxovid (NVX-CoV2373)
PCR: Polymerase chain reaction
Pf: Pfizer BioNTech Comirnaty (BNT162b2)
PICOBOO: Platform trial in COVID-19 priming and boosting
RAT: Rapid antigen test
REMAP-CAP: Randomized embedded multifactorial adaptive platform trial for community-acquired pneumonia
SARS-CoV-2: Severe acute respiratory syndrome coronavirus-2
SP: Safety population
VoC: Variants of concern
